# Assessing spirometry competence through certification in community‐based healthcare settings in Australia and New Zealand: A position paper of the Australian and New Zealand Society of Respiratory Science

**DOI:** 10.1111/resp.13987

**Published:** 2020-12-14

**Authors:** Irene Schneider, Leanne Rodwell, Sarah Baum, Brigitte M. Borg, Eleonora A. Del Colle, Emily R. Ingram, Maureen Swanney, Deborah Taylor, ANZSRS Community‐Based Spirometry Competency Working Group

**Affiliations:** ^1^ Respiratory Investigation Unit The Prince Charles Hospital Brisbane QLD Australia; ^2^ Department of Respiratory and Sleep Medicine Queensland Children's Hospital Brisbane QLD Australia; ^3^ Spirometry Training Company (Aust), Sessional Academic Charles Sturt University Sydney NSW Australia; ^4^ Respiratory Medicine The Alfred Hospital Melbourne VIC Australia; ^5^ School of Public Health and Preventive Medicine Monash University Melbourne VIC Australia; ^6^ Respiratory Laboratory Services, Department of Respiratory Medicine Box Hill Hospital Melbourne VIC Australia; ^7^ Pulmetrics Pty Ltd Melbourne VIC Australia; ^8^ Respiratory Physiology Laboratory Christchurch Hospital Christchurch New Zealand; ^9^ Respiratory Laboratory Hawke's Bay District Health Board, New Zealand Hawke's Bay New Zealand; ^10^ Spiro Me Training Hawke's Bay New Zealand

**Keywords:** certification, community health services, occupational health, quality control, spirometry

## Abstract

Spirometry has been established as an essential test for diagnosing and monitoring respiratory disease, particularly asthma and COPD, as well as in occupational health surveillance. In Australia and New Zealand, there is currently no pathway for spirometry operators in community‐based healthcare settings to demonstrate spirometry competence. The Australia and New Zealand Society of Respiratory Science (ANZSRS) has identified a need for developing a pathway for operators working in community‐based practices in Australia and New Zealand to demonstrate spirometry competence and certification. Spirometry certification provides evidence to patients, clients, employers and organizations that an individual has participated in an assessment process that qualifies them to perform spirometry to current international spirometry standards set out by the American Thoracic Society and the European Respiratory Society (ATS/ERS). This document describes a competence assessment pathway that incorporates a portfolio and practical assessment. The completion of this pathway and the award of certification confer an individual is competent to perform spirometry for 3 years, after which re‐certification is required. The adoption of this competency assessment and certification process by specialist organizations, and the commitment of operators performing spirometry to undergo this process, will enhance spirometry quality and practice in community‐based healthcare settings.

## Graphical abstract

**See related** Editorial

**See** cover image

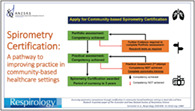

**Abbreviations**
ANZSRS: Australian and New Zealand Society of Respiratory Science
ARTP: Association for Respiratory Technology and Physiology
ATS/ERS: American Thoracic Society/European Respiratory Society
CBSC: Community‐Based Spirometry Competency
COPD: chronic obstructive pulmonary disease
QIP: Quality Innovation Performance

## CONTENTS

Introduction

The ANZSRS Community‐Based Spirometry Competency working group

The value of certification

Existing models of competence assessment

Pathway for certification in Australia and New Zealand

 Stage 1: Spirometry training and assessment

 Stage 2: Work experience and skill development

 Stage 3: Portfolio assessment

 Stage 4: Practical assessment

Applying for certification

 Initial application

 Re‐certification

 Resources and supporting documents

 Key performance indicators

Discussion

Conclusion

References

## INTRODUCTION

In 2017, the Australian and New Zealand Society of Respiratory Science (ANZSRS) published a position statement on the minimum requirements for spirometry training courses.^1^ The intention was to ensure spirometry courses available in Australia and New Zealand provided attendees with the theoretical and practical knowledge to meet the requirements for competency in spirometry.

The current paper is a supplement to the 2017 position statement and presents a process for assessing competency and awarding certification to trained operators performing diagnostic spirometry and occupational health screening spirometry in community‐based health settings. These may include, but are not limited to, the following: community and primary healthcare services, general practice, private practice specialist services, occupational health services, non‐tertiary hospitals, research facilities, pathology services, outreach services, allied health services and pharmacies.

Spirometry is performed in community‐based health services across the world. Despite its wide use, the quality of the results obtained in this setting does not always meet international practice standards.^2^, ^3^, ^4^ A review of spirometry undertaken for occupational health screening in Queensland, Australia, found that many operators lacked training and of those trained, the type and the format of training varied.^5^ The 2017 ANZSRS spirometry training position paper proposed a standardized approach to course content, structure and delivery.^1^ However, training alone, even when delivered to a standard, does not ensure ongoing good quality spirometry.^6^, ^7^

## THE ANZSRS COMMUNITY‐BASED SPIROMETRY COMPETENCY WORKING GROUP

The ANZSRS board recognized the need to improve the standard of spirometry in community‐based healthcare settings. Members of the special interest group, the ANZSRS Community‐Based Spirometry Competency (CBSC) working group, were selected by the board of the ANZSRS through an Expression of Interest process. All members of the CBSC working group recognized the importance of improving spirometry competence in community‐based healthcare settings and had extensive experience in developing, coordinating and facilitating spirometry training programmes in Australia and New Zealand. Several members were authors of the ANZSRS Spirometry Training position paper.^1^ The CBSC working group, referred to as the working group throughout the paper, developed the document on an honorary basis and received support from the ANZSRS board to convene regular teleconference meetings. Detailed information about the model, including a table of definition of terms used in this paper, is provided in Appendix S1 and S2 (Supplementary Information).

To address the needs of spirometry operators, physicians and the community, the CBSC working group has developed a model for assessing competence of spirometry operators in community‐based settings. The model has been derived following a review of current international and national processes for competence assessment in spirometry and other disciplines. The assessment tools presented in Supplementary Information include tools developed and used by the authors in various spirometry training programmes that align with the 2017 ANZSRS Spirometry Training position paper.

The aim of this position paper is to provide a model for assessing competence in spirometry. It also provides potential assessment tools for organizations that have the capability to implement and administer spirometry competency assessments in the future.

## THE VALUE OF CERTIFICATION

The working group agreed that the model for assessing competence would result in operator certification, which is dependent on the demonstration of spirometry competencies. For certification to be awarded, the candidate would demonstrate the application of spirometry knowledge and skills independent of the training environment. At the time of preparing this paper, there were no legal requirements for operators to be certified to perform spirometry in Australia and New Zealand.

Certification is a method used to provide confidence that an individual possesses the skills and knowledge to perform their job to current practice standards.^8^ The aim of certification is to benchmark skills against a standard and add value to an individual's professional development. In jobs where there is a high risk of harm to the individual performing the job, or others, certification becomes a legal obligation.^8^

Education and training set a minimum standard of practice, whereas certification recognizes an individual's knowledge and expertise in a field. For example, in Californian hospitals where leaders are certified in infection prevention and control, there are lower rates rate of methicillin‐resistant *Staphylococcus aureus* (MRSA) infection when compared to staff who are not certified for the task.^9^

## EXISTING MODELS OF COMPETENCE ASSESSMENT

Limited models for assessing spirometry competency in community‐based services exist. Many of the elements of these programmes were reviewed by the working group for broad principles that could be adopted. In the UK, the Association for Respiratory Technology and Physiology (ARTP) certifies operators for 3 years at one of the three different levels: performing, performing and interpreting or only interpreting spirometry tests.^10^ The Canadian Nurses Association (CNA) offers an Occupational Health Certification that includes spirometry as a specialty area.^11^ The Canadian Association of Cardio‐Pulmonary Technologists offers certification for allied health practitioners, including registered nurses, with a 3–5‐year re‐certification period. The United States National Institute for Occupational Safety and Health (NIOSH) recognizes approved spirometry training course certificates for 5 years, but does not award certification in spirometry.^12^ The Harmonized Education of Respiratory Medicine in European Specialties (HERMES) project framework was adopted to develop resources for standardized spirometry training, assessment and certification across Europe.^13^ In Queensland, Australia, the Coal Mine Workers Health Scheme (CMHWS) requires spirometry operators to complete Quality Innovation Performance (QIP) accredited spirometry training programmes for CMWHS employment medicals, followed by refresher training 1 year after the initial training and every 3 years thereafter.^14^ A current model exists for recognizing competence in thoracic ultrasound in Australia and New Zealand.^15^ Even though this process has been established for a different procedure, elements of the programme align with the model proposed for spirometry in this paper.

There is a sparsity of literature regarding the uptake of formal competency recognition and its impact on spirometry quality. The National Health Service (NHS) in the UK, in collaboration with the ARTP have introduced a national health register for spirometry performance and interpretation.^16^ The deadline for all operators to comply with this registration is March 2021. Although not mandatory, it is a best practice recommendation and non‐compliance may lead to issues with the Care Quality Commission, the independent regulator of health and social care in the UK.^17^ Robust evidence for the effect of mandatory competence assessment on the quality of spirometry testing will only be available once the processes are implemented and evaluated.

## PATHWAY FOR CERTIFICATION IN AUSTRALIA AND NEW ZEALAND

The responsibility for awarding spirometry certification in recognition of competence is the role of specialist organizations, referred to as the ‘certifiers’ in the paper. The certifier will have the resources to receive applications for certification, receive uploaded assessment evidence and outcomes, maintain a database of certified operators and qualified assessors, field enquiries, provide support to operators and assessors and send reminders for re‐certification.

**Figure 1:**
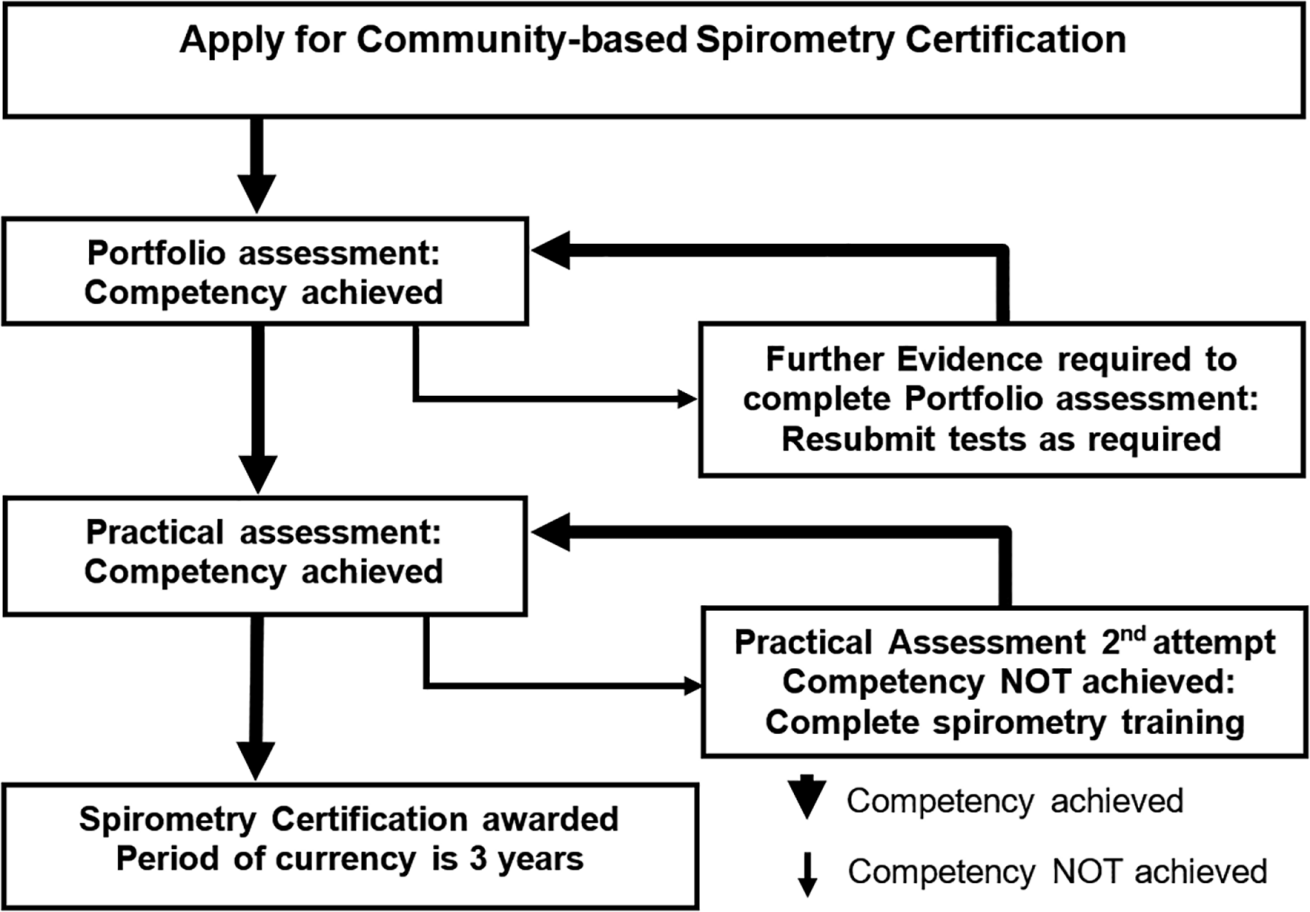
Pathway for assessing competence and attaining certification in spirometry practice

The proposed model for certifying operators is outlined in Figure 1. There are four stages to achieving competence and certification which are detailed below. In developing the model best practice guiding principles of reliability, flexibility, validity and fairness of assessment were considered.

The model is flexible and fair in that evidence can be collected through a variety of pathways. Assessment tools, used by the authors in existing spirometry training programmes, have been reviewed by the working party for use in this pathway. Although these tools have not been formally evaluated, the working party is confident that they are valid and reliable, by virtue of their current use to assess participants completing established, and in some instances QIP accredited, spirometry training programmes in Australia and New Zealand. Extensive discussion by working group members has resulted in the development of assessment tool templates recommended for use by the certifier to adapt as required. The assessment tools; information for certifiers, candidates and assessors; and further information about the competency framework are provided in Supplementary Information.

Candidates for certification will be assessed against specified spirometry competency elements and performance criteria by independent assessors as outlined in Appendix S7 (Supplementary Information). Both a portfolio and practical assessment (Appendices S1–S8 in Supplementary Information) are included in the certification pathway. A list of independent assessors will be provided by the certifier.

### Stage 1: Spirometry training and assessment

Candidates are advised to complete a spirometry training programme that includes the mandatory components as outlined in the ANZSRS spirometry training position statement.^1^ The content requirements of training courses are based on the American Thoracic Society/European Respiratory Society (ATS/ERS) standards for spirometry.^3^, ^4^ Spirometry training provides base level skills and knowledge. More importantly, training that incorporates a portfolio assessment tool provides the opportunity for skills to be applied and consolidated in the workplace. An experienced operator may apply for certification without having completed recent training; however, further training will be advised during the process if there is evidence of unsafe practice.

### Stage 2: Work experience and skill development

Workplace experience builds the candidate's confidence and experience in performing quality spirometry. It is recommended that candidates undergo a period of work‐based practice after completion of a spirometry training programme before applying for certification. Candidates will be advised to maintain a logbook of spirometry tests performed in the workplace which would be validated by a workplace supervisor or mentor and provide evidence of ongoing spirometry practice. The supervisor need not be experienced in spirometry but should be able to confirm the number of spirometry tests performed by the candidate. There is no evidence to support a defined minimum number of tests that operators must perform to attain and maintain competence, although a positive correlation between acceptability and frequency of tests performed by an operator has been shown in one study by Bellia *et al*.^18^ Based on observations by spirometry trainers and consensus by the working group, it is not unreasonable to expect that a minimum of 100 spirometry tests per year be performed by spirometry operators who had achieved initial competence.

### Stage 3: Portfolio assessment

Candidates will be required to provide a portfolio of a minimum of 10 client tests performed in the workplace, including an analysis of test quality, interpretation and quality control measures consistent with the current ATS/ERS standards and guidelines (Appendices S6,S8,S9 in Supplementary Information) and spirometry competency elements (Appendix S7 in Supplementary Information). If the candidate has completed a training programme that includes a workplace portfolio assessment, then this may provide the evidence required for the portfolio submission. If competence is not demonstrated in the submitted portfolio, then the candidate will submit further tests until 10 are completed to the required standard. The re‐submission provides opportunity for learning from the feedback provided to the candidate. Successful completion of the portfolio will allow the candidate to progress through to the practical assessment.

### Stage 4: Practical assessment

Candidates will be required to undergo a practical assessment (Appendices S6,S8,S10 in Supplementary Information). The practical assessment will evaluate the candidate's ability to perform spirometry with a client in the workplace consistent with the current ATS/ERS standards and guidelines and spirometry competency elements (Appendix S7 in Supplementary Information). The candidate will also be required to demonstrate application of established quality assurance, quality control and infection control procedures. To provide accessibility to rural and remote candidates, the practical assessment may be conducted through a virtual platform instead of a physical face‐to‐face observation.

## APPLYING FOR CERTIFICATION

### Initial application

The candidate will apply for certification with the certifier. Detailed instructions and description of the evidence required by the certifier will be made available to the candidate. The evidence required to recognize spirometry competence will include:

1. Certificate of completion of spirometry training (if recently completed) and a logbook of the number of spirometry tests performed in the workplace.
2. Portfolio assessment completion (if part of a recently completed spirometry training) or portfolio of test results (if not completed as part of spirometry training).
3. Practical assessment completion.

The evidence will be submitted to the certifier by the candidate. If the evidence provided is sufficient to recognize the candidate as competent to perform spirometry to current standards,^4^ then the candidate will be awarded certification for a period of 3 years.

### Re‐certification

Spirometry operators will need to be re‐certified every three years. The evidence required to show maintenance of competence may include a spirometry portfolio, record of frequency of practice, spirometry refresher training certificate, spirometry auditing or other evidence deemed appropriate by the certifier.

### Resources and supporting documents

Examples of resources for use by the certifier, the candidate and assessors are found in Appendices S9 and S10 (Supplementary Information). These include information for candidates and assessors (Appendices S4–S6 in Supplementary Information), including assessment requirements and performance evidence, spirometry competency elements and performance criteria, spirometry portfolio cover sheet and a practical assessment checklist.

### Key performance indicators

The ATS and ERS provide clear guidelines and recommendations for grading spirometry test quality.^4^, ^19^ The defined A–F grades provide interpreters with additional confidence in the test results achieved by patients in each testing session. Key performance indicators for assessing the quality of test results submitted by certification candidates will utilize this grading system. However, evidence to assess competence of an operator, based on a percentage cut‐off for compliance with international standards, is lacking. Several studies utilized a quality rating system to show compliance with international standards in primary care^7^ and lung function laboratories.^20^ Levels of compliance were >87% and >90%, in primary care services and laboratories, respectively, following various interventions (feedback, training, re‐education, auditing and mentoring). On the basis of these findings, it would not be unreasonable to expect that the criteria for achieving competence in spirometry operators in community‐based healthcare settings would require that >80% of submitted test results achieve an A or B grade. Further studies to validate this recommendation are needed.

## DISCUSSION

The working group encountered several obstacles during the development of the certification model. First, there is limited evidence to recommend a model for spirometry competency assessment and certification in Australia and New Zealand. To this end, the proposed pathway is a result of extensive discussions and consensus by the working group, who have recognized expertise in spirometry practice, training and assessment in Australia and New Zealand. Second, the proposed model is not yet supported by a specialist organization, that is the certifier, so solutions to implementation problems, arising during working group discussions, were purely hypothetical. Consequently, the working group felt that a flexible pathway may better align with potential certifiers' existing resources and infrastructure. Third, resources used in established spirometry training programmes were collated to produce the assessment tools, and the working party deemed the developed tools were sufficiently robust to assess competence. However, a formal evaluation of the tools will provide confidence to all stakeholders that the certification model is able to deliver its intended objectives. Lastly, although all spirometry operators will have access to the certification pathway, it is a voluntary process not anticipated to be mandated in the near future.

## CONCLUSION

This document outlines a model for certification of operators performing spirometry in community‐based practices in Australia and New Zealand. The model allows operators to demonstrate skills and knowledge previously gained through training and workplace practice. Certification will provide confidence to employers, patients and clients that an operator is competent to perform spirometry to current international practice standards. Currently, few programmes exist against which to benchmark the proposed pathway to achieving spirometry competence, and this is a limitation. Future work will include developing key performance indicators against which to measure the success of the pathway to certification and to inform future improvements.

## Disclosure statement

The authors (I.S., L.R., S.B., B.M.B., E.A.D.C., E.R.I., D.T. and M.S.) are facilitators and assessors in spirometry training programmes that provide a service for fees or through government funding. Income generated from fees covers the costs incurred in training and assessing course participants and related to that training only. The authors or the organizations they work for have the potential to earn income as assessors on a fee for service basis should the certification process be established.

## Supporting information


**Appendix** **S1.** Definition of terms and abbreviations.
**Appendix S2.** Overview of certification framework.
**Appendix S3.** Standards for performing spirometry.
**Appendix S4.** Information for candidates.
**Appendix S5.** Information for assessors.
**Appendix S6.** Assessment requirements and performance evidence.
**Appendix S7.** Spirometry competency elements and performance criteria.
**Appendix S8.** Competency elements: portfolio and practical assessments.
**Appendix S9.** Spirometry portfolio resources.
**Appendix S10.** Spirometry practical resources.
**Appendix S11.** References.

**Visual Abstract** Spirometry Certification: A pathway to improving practice in community‐based healthcare settings.

## References

[1] Swanney MP , O'Dea CA , Ingram ER , Rodwell LT , Borg BM . Spirometry training courses: content, delivery and assessment – a position statement from the Australian and New Zealand Society of Respiratory Science. Respirology 2017; 22: 1430–5. 28681980 10.1111/resp.13133

[2] Miller MR , Crapo R , Hankinson J , Brusasco V , Burgos F , Casaburi R , Coates A , Enright P , van der Grinten CPM , Gustafsson P *et al* General considerations for lung function testing. Eur. Respir. J. 2005; 26: 153–61. 15994402 10.1183/09031936.05.00034505

[3] Miller MR , Hankinson J , Brusasco V , Burgos F , Casaburi R , Coates A , Crapo R , Enright P , Van der Grinten CPM , Gustafsson P *et al* Standardisation of spirometry. Eur. Respir. J. 2005; 26: 319–38. 16055882 10.1183/09031936.05.00034805

[4] Graham BL , Steenbruggen I , Miller MR , Barjaktarevic IZ , Cooper BG , Hall GL , Hallstrand TS , Kaminsky DA , McCarthy K , McCormack MC *et al* Standardization of spirometry 2019 update. An official American Thoracic Society and European Respiratory Society technical statement. Am. J. Respir. Crit. Care Med. 2019; 200: e70–88. 31613151 10.1164/rccm.201908-1590ST PMC6794117

[5] Monash Centre for Occupational and Environmental Health . Review of Respiratory Component of the Coal Mine Workers' Health Scheme for the Queensland Department of Natural Resources and Mines. 2016 [Accessed 13 Feb 2018.] Available from URL: https://www.dnrm.qld.gov.au/__data/assets/pdf_file/0009/383940/monash-qcwp-final-report-2016

[6] Burton MA , Burton DL , Simpson MD , Gissing PM , Bowman SL . Respiratory function testing: the impact of respiratory scientists on the training and support of primary healthcare providers. Respirology 2004; 9: 260–4. 15182279 10.1111/j.1440-1843.2004.00563.x

[7] Borg BM , Hartley MF , Fisher MT , Thompson BR . Spirometry training does not guarantee valid results. Respir. Care 2010; 55: 689–94. 20507650

[8] Australian Government . Department of Education, Skills and Employment. HLTHPS004 Measure Spirometry. 2020 [Accessed 12 May 2020.] Available from URL: https://training.gov.au/TrainingComponentFiles/HLT/HLTHPS004_R1.pdf

[9] Porgorzelska M , Stone PW , Larson ELL . Certification in infection control matters: impact of infection control department characteristic and policies on rates of multidrug‐resistant infection. Am. J. Infect. Control 2012; 40: 96–101. 22381222 10.1016/j.ajic.2011.10.002 PMC3329760

[10] Association for Respiratory Technology and Physiology (ARTP) . Spirometry Certification. [Accessed 12 Nov 2019.] Available from URL: https://www.artp.org.uk/Spirometry-Certification/

[11] Alberta Occupational Health Nurses Association (AOHNA) . CAN Certification Program. [Accessed 27 June 2018.] Available from URL: http://aohna.org/resources-links/certification-information

[12] Centers for Disease Control and Prevention , The National Institute for Occupational Safety and Health (NIOSH), Spirometry Training Program. [Accessed 15 May 2020.] Available from URL: https://www.cdc.gov/niosh/topics/spirometry/training.html

[13] Steenbruggen I , Mitchell S , Severin T , Palange P , Cooper BG ; HERMES Task Force . Harmonising spirometry education with HERMES: training a new generation of qualified spirometry practitioners across Europe. Eur. Respir. J. 2011; 37: 479–81. 21357919 10.1183/09031936.00187810

[14] Thoracic Society of Australia and New Zealand . Standards for the delivery of spirometry for coal mine workers. 2017 [Accessed 25 May 2020.] Available from URL: https://www.dnrme.qld.gov.au/__data/assets/pdf_file/0003/1274421/tsanz-spirometry-standards.pdf

[15] Williamson JP , Twaddell SH , Lee GYC , Salamonsen M , Hew M , Fielding D , Nguyen P , Steinfort D , Hopkins P , Smith N *et al* Thoracic ultrasound recognition of competence: a position paper of the Thoracic Society of Australia and New Zealand. Respirology 2017; 22: 405–8. 28102968 10.1111/resp.12977

[16] NHS England and NHS Improvement . Spirometry commissioning guidance. [Accessed 23 Oct 2020.] Available from URL: https://www.england.nhs.uk/wp-content/uploads/2020/03/spirometry-commissioning-guidance.pdf

[17] Association for Respiratory Technology and Physiology . Spirometry Certification. [Accessed 23 Oct 2020.] Available from URL: https://www.artp.org.uk/Spirometry-Register

[18] Bellia V , Pistelli R , Catalano F , Antonelli‐Incalzi R , Grassi V , Melillo G , Olivieri D , Rengo R . Quality control of spirometry in the elderly, the SA.R.A. study. Am. J. Respir. Crit. Care Med. 2000; 161: 1094–100. 10764296 10.1164/ajrccm.161.4.9810093

[19] Culver BH , Graham BL , Coates AL , Wanger J , Berry CE , Clarke PK , Hallstrand TS , Hankinson JL , Kaminsky DA , MacIntyre N *et al* Recommendations for a standardized pulmonary function report. Am. J. Respir. Crit. Care Med. 2017; 196: 1463–72. 29192835 10.1164/rccm.201710-1981ST

[20] Borg BM , Hartley MF , Bailey MJ , Thompson BR . Adherence to acceptability and repeatability criteria for spirometry in complex lung function laboratories. Respir. Care 2012; 57: 2032–8. 22709916 10.4187/respcare.01724

