# Supplementary material for: Assessing spirometry competence through certification in community‐based healthcare settings in Australia and New Zealand: A position paper of the Australian and New Zealand Society of Respiratory Science
Source: Respirology. 2020 Dec 14;26(2):147–52. doi: 10.1111/resp.13987 (PMC7898916; doi:10.1111/resp.13987)
Supplement: Supplementary file 1 — Appendix S1. Definition of terms and abbreviations. Appendix S2. Overview of certification framework. Appendix S3. Standards for performing spirometry. Appendix S4. Information for candidates. Appendix S5. Information for assessors. Appendix S6. Assessment requirements and performance evidence. Appendix S7. Spirometry competency elements and performance criteria. Appendix S8. Competency elements: portfolio and practical assessments. Appendix S9. Spirometry portfolio resources. Appendix S10. Spirometry practical resources. Appendix S11. References. [file RESP-26-147-s001.docx]

**SUPPLEMENTARY INFORMATION**

**Assessing spirometry competence through certification in community-based healthcare settings in Australia and New Zealand: A position paper of The Australian and New Zealand Society of Respiratory Science**

Irene Schneider ^1,2^ BSc (Hons), Grad Cert (Higher Educational Studies), CERT IV (Training and Assessment)

Leanne Rodwell ^2^ PhD, BSc, Grad Dip Phty, CRFS

Sarah Baum ^3^ BSc (Nutrition), Post Grad Cert (Resp. Sc)

Brigitte M Borg ^4,5^ BAppSc (Dist), CRFS

Eleonora Alice Del Colle ^6^ BAppSc, CRFS

Emily Rose Ingram ^7^ BSc (Hons), Grad Cert, CRFS

Maureen Swanney^†^ ^7^ PhD, MSc (Hons), BSc, FThorSoc, CRFS

Deborah Taylor ^8^ HNC/BTEC (Medical Physics and Physiological Measurement), PGCertMedTech, CRFS

On behalf of the ANZSRS Community-Based Spirometry Competency Working Group

^†Deceased February 17, 2019^

^1^ Respiratory Investigation Unit, The Prince Charles Hospital, Brisbane, QLD, Australia;

^2^ Department of Respiratory and Sleep Medicine, Queensland Children’s Hospital, Brisbane, QLD, Australia

^3^ Spirometry Training Company (Aust); Sessional Academic, Charles Sturt University

^4^ Respiratory Medicine, The Alfred Hospital, Melbourne, VIC, Australia

^5^ School of Public Health & Preventive Medicine, Monash University, Melbourne, Australia

^6^ Respiratory Laboratory Services, Department of Respiratory Medicine, Box Hill Hospital, Box Hill, Victoria, Australia; Pulmetrics Pty Ltd

^7^ Respiratory Physiology Laboratory, Christchurch Hospital, Christchurch, New Zealand

^8^ Respiratory Laboratory, Hawke's Bay District Health Board, New Zealand; Spiro Me Training, Hawke’s Bay, New Zealand

Appendix S1 Definition of terms and abbreviations

Appendix S2 Overview of certification framework

Appendix S3 Standards for performing spirometry

Appendix S4 Information for candidates

Appendix S5 Information for assessors

Appendix S6 Assessment requirements and performance evidence

Appendix S7 Spirometry competency elements and performance criteria

Appendix S8 Competency elements - portfolio and practical assessments

Appendix S9 Spirometry portfolio resources

Appendix S10 Spirometry practical resources

Appendix S11 References

**Appendix S1. Definition of terms and abbreviations**

| ANZSRS | Australian and New Zealand Society of Respiratory Science |
| --- | --- |
| Assessments | Includes portfolio and practical assessment |
| Assessor | Individuals deemed competent by the certifying body to assess the Candidate |
| ATS/ERS | American Thoracic Society/European Respiratory Society |
| Candidate | Individuals performing spirometry in the community and applying for certification |
| Certifier | A specialist organisation that has resources to receive applications for certification, receive uploaded assessment evidence and outcomes, maintain a database of certified operators and qualified Assessors, field enquiries and provide support to operators and Assessors |
| Client | This term includes patients in a health setting, an employee as part of pre- employment or occupational screening, a research subject, a participant in a rehab program, and any other person undergoing spirometry in a health setting |
| Competency | A set of knowledge, skills and attitudes being assessed against learning outcomes and industry standards, e.g. the Spirometry Competency |
| Competence | Quality of the Candidate in demonstrating the knowledge, skills and attitudes that enables them to test independently to an industry standard |
| Competency elements | A broad description of the work activities, skills and tasks that a Candidate is required to perform independently to be assessed as competent |
| Community-based spirometry | Spirometry performed in General Practices, Pharmacies, Community Health Centers, and Workplaces in Australia and New Zealand (excluding Respiratory Laboratories) |
| FEV_1_ | Forced Expiratory Volume in 1 second |
| FVC | Forced Vital Capacity |
| PEF | Peak Expiratory Flow |
| Performance Criteria | A detailed description of the work activities in each competency element |
| Performance Evidence | The type of evidence or cues used to illustrate the performance criteria. In this framework the evidence is obtained from the practical and portfolio assessment |
| QC | Quality Control |
| QA | Quality Assurance |
| Spirometry Portfolio Assessment coversheet | A checklist for both Candidates and Assessors against which the portfolio is produced and assessed |

**Appendix S2. Overview of certification framework**

This guide outlines the process for attaining competence and certification in performing spirometry including short-acting bronchodilator responsiveness, if applicable. Candidates who perform post-bronchodilator responsiveness testing in their workplace will indicate in their application if they need to include this competency in the assessment.

There are two components to the competence process:

1. Portfolio assessment

2. Practical assessment

The successful completion of these assessments will provide the evidence that competence has been achieved in the workplace.

The certifying body will be required to:

- Provide a summary of the outcomes of the competence assessment to Candidates
- Provide feedback to the Candidate if further evidence is required
- Issue certification certificates to Candidates including date of certification and period of currency
- Maintain a database of Certified operators and approved Assessors.

The resources supplied in this supplement can be adapted for electronic formats, for example, a portfolio coversheet may be an electronic form to which a client test report is attached as evidence.

**Appendix S3. Standards for performing spirometry**

### The spirometry competence requirements defined in this document are based on current American Thoracic Society and European Respiratory Society standards (ATS/ERS) ^1^.

In addition, Candidates will be required to provide the following local workplace protocols and procedures as evidence of adherence to:

- Local medical emergency procedures
- Occupational health and safety procedures
- Infection control procedures
- Obtaining (and documenting) informed verbal or implied consent for test
- Short-acting bronchodilators administration procedures

**Appendix S4. Information for Candidates**

Prior to commencing the competence assessment and certification process, the Candidate must read and understand all the requirements for achieving competence as outlined in this document and seek clarification from the Certifier or the Assessor if needed.

Candidates must complete all the designated tasks and assessments as outlined in this document. The Candidate must submit the completed evidence to the Certifier in a format specified by the Certifier.

The Candidate must submit evidence of prior completion of a spirometry training program and experience in spirometry practice, for example in the form of a logbook. Further spirometry training may be recommended if the Candidate is deemed not competent during any stage of the certification process.

The timelines for completion of individual components of the certification process should be set after discussion between the Candidate and the Assessor. In general, it is suggested that the competency assessment is completed within 12 months of initial certification application.

In summary, Candidates must:

- Read and understand all documents related to competence attainment and certification.
- Seek clarification from the Certifier about any aspect of the competence process.
- Conduct client testing independently for portfolio and practical assessments.
- Demonstrate all competency elements as outlined in Section 7.
- Submit all assessments to the Assessor (or Certifier, if required) in a timeframe as determined by the Assessor (or Certifier)

**Appendix S5. Information for Assessors**

This document must be read and understood by all Assessors. The Assessor will be impartial and fair in their role in assessing the Candidate’s skills and knowledge within this assessment framework.

The Assessor will be required to:

- Assess the evidence provided by the Candidate using standardized portfolio and practical assessment tools as supplied by the Certifier.
- Complete all assessment documentation with comments and assessment outcomes, providing constructive feedback where required.
- Return completed documentation to the Candidate (and/or the Certifier as per Certifier’s requirements).

**Appendix S6. Assessment requirements and performance evidence**

- 1. **Portfolio Assessment**

The portfolio assessment will evaluate the Candidate’s application of skills and knowledge in the workplace consistent with current ATS/ERS standards and guidelines and the competency elements listed in S7. Specific areas of competency covered by the portfolio assessment are outlined in S8. The S*pirometry Portfolio Assessment coversheet* (S9) will be used to assess competence. The coversheet must be fully completed by the Candidate for each test prior to submitting the portfolio.

The portfolio will include:

1. 10 spirometry tests performed by the Candidate covering a range of clients that best reflect the workplace context. For example:

- Adult and paediatric clients
- Normal spirometry
- Obstructive pattern spirometry
- Restrictive pattern spirometry
- Mixed obstructive/restrictive pattern spirometry
- Spirometry with and without significant bronchodilator responsiveness
- Spirometry from a client that was difficult to test to show troubleshooting skills. Any unacceptable tests (maximum 2) must include detailed technical comments outlining why they are unacceptable

1. Each client test report will be accompanied by a completed *Spirometry Portfolio Assessment Coversheet* (S9) which will include the following details:

- spirometer make, model and software version
- calibration syringe brand, model and date of most recent validation
- reference equations used
- indication for testing
- a brief description of the workplace i.e. geographical location, testing environment, client population.
- a description of the routine infection control process
- a description of quality assurance processes including equipment calibration and/or calibration verification

1. Each client test report will be de-identified and include:

- client’s date of birth, age, height, weight, birth sex, ethnicity
- for each trial attempt irrespective of whether acceptability, usability and/or repeatability criteria have been met:
- graphical presentation of each of the trials performed (including both flow-volume and time – volume curves)
- data for all trials attempted (FEV_1_, FVC, FEV_1_/FVC %, PEF)
- time of test
- final results including identification of spirometry pattern
- a technical comment on test quality assessing the following criteria: acceptability, repeatability, usablility and including a quality ^-^grading for FEV_1_ and FVC ^1^
- identification of the spirometry pattern
- test results that clearly identify the Candidate as conducting the test
- if conducted, assessment of bronchodilator responsiveness test results quantified for percentage and volume, where positive.

1. Evidence of quality assurance activities will include:

- calibration and/or calibration verification records from the spirometer used on the day of testing
- checks at multiple flows (low, medium and high) for flow-based spirometers
- action taken if there are calibration or calibration verification problems.

*Information for Portfolio Assessors*

The *Spirometry Portfolio Assessment Coversheet* (S9) will be completed by the Candidate. It provides evidence of successful completion of the portfolio, and that the evidence provided is valid, authentic, reliable and current. Specifically, the following criteria will be required to assess the portfolio:

- Correct number of tests submitted
- Completed cover sheet
- Evidence of own work
- Correct time frame
- Covers the range of client types suggested above
- Evidence of skills and knowledge to produce and evaluate valid and reliable data as outlined by the coversheet.

The Assessor must include enough information in the Assessor’s comments section to support the final assessment outcome.

- 1. **Practical Assessment**

The practical assessment will evaluate the Candidate’s application of skills and knowledge in the workplace consistent with current ATS/ERS standards and guidelines. The Candidate will also be required to follow established quality assurance, quality control, infection control and safety procedures to complete the tasks outlined in the Competency Elements Performance Criteria in S7. Specific areas of competency covered by the practical assessment are outlined in S8.

The S*pirometry Practical Assessment checklist* (S10) will be used to assess competence, through observation, verbal questioning and written responses.

The Candidate will be asked to demonstrate the following competencies (S7):

- Prepare the testing environment and equipment
- Prepare the client
- Perform baseline spirometry
- Complete a spirometry test
- Produce a spirometry report

Candidates performing post bronchodilator spirometry in their workplace will be asked to demonstrate the following competency (S7) in addition to those above:

- Perform post bronchodilator spirometry

During the practical assessment the Candidate will also be required to demonstrate:

- an understanding of the spirometry test, the key parameters measured (FEV_1_, FVC, FEV_1_/FVC, PEF) and the graphs generated during testing
- an understanding of the risks associated with spirometry testing
- the ability to troubleshoot maneuvers and patient related problems
- the ability to troubleshoot equipment related problems if they occur
- the ability to manage spirometer calibration and/or calibration verification failure
- the ability to apply infection control principles before, during and after the testing session.

*Information for Practical Assessors*

Prior to the practical assessment, the Assessor will ensure that the Candidate is familiar with the relevant assessment documents and understands the assessment process, including the specific assessment criteria. They will liaise with the Candidate regarding the date, time, location and method (if occurring remotely) of the assessment. Finally, the Assessor will discuss the outcomes of the assessment with the Candidate and provide the Candidate with verbal and/or written feedback.

- 1. **Re-assessment Process**

Figure 1 provides an outline of the certification process. A Candidate should only apply for certification if confident they can successfully complete the process. If spirometry assessments are not successfully completed, or the evidence provided does not meet the standard, then the Candidate will be provided with the opportunity to re-sit or resubmit the assessments. If the Candidate is unsuccessful on resubmission, they will be required to complete further training or workplace practice before re-applying for certification.

**Appendix S7. Spirometry competency elements and performance criteria**

Competence will be assessed against the competency elements and performance criteria listed below. (Extracted from The Statewide Clinical Measurements Network, Queensland Health: Griffith University Clinical Measurements, Competencies, Key Learning Objectives and tools for Assessing Independent Clinical Measurement Scientist Practice. A report commissioned by Allied Professions of Queensland (AHPOQ). 2014. Unpublished document).

1. Prepare the testing environment and equipment

- Ensure appropriate workplace conditions for test (controlled environment, including room temperature)
- Prepare testing environment (infection control and prevention procedures)
- Prepare spirometer for testing (calibration/calibration verification)
- Ensure selection of appropriate reference values (e.g., GLI 2012) ^2,3^
- Operate test equipment according the manufacturer’s recommendations and workplace protocols

1. Prepare the client

- Review referral document or reason for testing and relevant client information
- Confirm client identity to ensure correct client for spirometry procedure
- Identify any contraindications for testing^1^
- Measure client’s height (essential for spirometry) and weight (if possible)
- Record correct demographic details (age, date of birth, height, sex, ethnicity, and weight, if possible)
- Record recent respiratory medications
- Provide clear and comprehensible description of the nature and purpose of the test

1. Perform baseline spirometry

- Perform test in accordance with accepted guidelines and safe working practices
- Maintain communication with the client throughout the procedure
- Ensure correct client technique (including posture, use of nose-peg)
- Assess client for adverse events related to the test
- Produce technically acceptable and repeatable test results

1. Perform bronchodilator responsiveness testing

- Perform test as requested in accordance with accepted guidelines and safe working practices
- Administer bronchodilator according to workplace protocols
- Wait the required time after the bronchodilator has been given before testing
- Maintain communication with the client throughout the procedure
- Ensure correct client technique (including posture, use of nose-peg)
- Assess client for adverse events related to the test
- Produce technically acceptable and repeatable test result

1. Complete spirometry test

- Dispose of disposable materials as per organizational procedures
- Clean and store test equipment and testing environment according to accepted guidelines and organizational protocols

1. Produce a spirometry report

- Generate a suitable report including correct client information
- Select best values to ATS/ERS criteria
- Include technical comments regarding acceptability, repeatability and usability^1^, of results and identifying cause of suboptimal test performance
- Grade the quality of the test to current ATS/ERS recommendations ^1^
- Identify spirometry pattern and severity ^4^

**Appendix S8. Competency elements - in portfolio and practical assessments**

Competency Elements and Performance Criteria assessed by the practical and portfolio assessments are summarized in Table S8. These assessments will provide the evidence required to demonstrate the Performance Criteria within each Competency Element detailed in S7.

**Table S8.** Summary of mandatory Competency Elements and Performance Criteria assessed by the practical and portfolio assessments.

| **Competency elements** | **Practical** | **Portfolio** | **Competency elements** |
| --- | --- | --- | --- |
| **General** | | | |
| Demonstrate an understanding of the principles of the test | ✓ |  | 7.1-7.6 |
| **Preparing the testing environment, equipment and client** | | | |
| Ensure appropriate workplace conditions for test | ✓ |  | 7.1 |
| Apply relevant infection control procedures | ✓ |  | 7.1, 7.5 |
| Assemble and operate test and related equipment | ✓ |  | 7.1 |
| Calibrate and/or verify calibration of test equipment using 3-L syringe | ✓ | ✓ | 7.1 |
| Check appropriate reference values | ✓ | ✓ | 7.1 |
| State indications and contraindications for testing | ✓ | ✓ | 7.2 |
| Identify client and record client demographics and recent respiratory medications | ✓ | ✓ | 7.2 |
| Provide clear explanation of test procedure | ✓ |  | 7.2 |
| **Performing the test** | | | |
| Ensure correct client technique | ✓ | ✓ | 7.3 |
| Perform test in accordance with current guidelines and safe working practices (including bronchodilator responsiveness testing) | ✓ |  | 7.3, 7.4 |
| Demonstrate knowledge of testing criteria ^1^ (number of trials, within- and between-manoeuvre evaluation, acceptability, repeatability and usability criteria) | ✓ | ✓ | 7.3, 7.4 |
| Assess client for adverse events | ✓ |  | 7.3, 7.4 |
| Apply appropriate troubleshooting strategies (client and equipment) | ✓ | ✓ | 7.3, 7.4 |
| **Completing the test and producing a report** | | | |
| Prepare equipment for shutdown, clean-up and storage and disposal of consumables | ✓ |  | 7.5 |
| Generate a report including correct client information, selection of best values, technical comments and test session quality grading | ✓ | ✓ | 7.6 |
| Identify spirometry pattern | ✓ | ✓ | 7.6 |

**Appendix S9. Spirometry portfolio requirements and coversheet**

The purpose of this assessment is to evaluate the Candidate’s ability to perform spirometry on a client in the workplace with an emphasis on consistent best practice. This assessment will focus on workplace practice and transfer of skills. Prior to commencing the Spirometry Portfolio assessment, ensure that you have read and understood the following sections of this document:

- Portfolio Assessment instructions (S6.1)
- Competency elements and performance criteria for spirometry (S7)
- Spirometry portfolio assessment coversheet (S9)

If a spirometry portfolio has been successfully completed as part of recent spirometry training, then this can be provided as evidence of completion of a portfolio for the certification process. If it does not meet the requirements of the certification process, then the Candidate will need to provide a portfolio complying with the portfolio assessment requirements outlined in S6.1.

### Table S9.1 Spirometry Portfolio Assessment Coversheet

| Candidate name: ___________________________________________________ | | | |
| --- | --- | --- | --- |
| Portfolio Case #: _____________________ | | | Test Date: ____________________ |
| Spirometer make and model and software version: _________________________ | | | |
| 3-L Calibration Syringe brand: | ___________________________________ | | |
| Syringe calibration verification date: | ___________________________________ | | |
| Geographical location of testing: _______________________________________ | | | |
| Routine infection control process (brief description):  __________________________________________________________________________________________________________________________________ | | | |
| Routine quality control process (brief description):  __________________________________________________________________________________________________________________________________ | | | |
| **Demographic details of client:** | | | |
| Date of Birth: ____________________ | | Age (years): ______________________ | |
| Birth Sex: Male/Female | | Height (cm): ______________________ | |
| Weight (kg): _____________________ | | Ethnicity: ________________________ | |
| Reference equations used: ___________________________________________ | | | |
| Indications for spirometry testing:_______________________________________ _________________________________________________________________ | | | |

| **Assessment of spirometry test** | | **Candidate to complete**  **Yes/No** | | **Assessor’s comments**  *Provide enough information to support the final assessment outcome* | |
| --- | --- | --- | --- | --- | --- |
| Have the ambient conditions (e.g. temperature) been updated on the spirometer on the day of this test? | |  | |  | |
| Has the calibration and /or calibration verification passed on the day of this test? | |  | |  | |
| Date of most recent calibration and/or calibration verification:_________________ | | | | | |
| Have the client’s demographic details been entered correctly into the spirometer? | |  | |  | |
| Do the following trials meet ATS/ERS acceptability criteria? Where trials were unacceptable, what error(s) were identified? | | | | | |
| Trial # | Candidate’s comment | | |  | |
| Trial 1 |  | | |  | |
| Trial 2 |  | | |  | |
| Trial 3 |  | | |  | |
| Trial 4 |  | | |  | |
| Trial 5 |  | | |  | |
| Trial 6 |  | | |  | |
| Trial 7 |  | | |  | |
| Trial 8 |  | | |  | |
| Did the client achieve 3 technically acceptable trials? | |  | |  | |
| If not, has an appropriate technical comment detailing the problems been added to the report? | |  | |  | |
| Does FEV_1_ meet ATS/ERS repeatability criteria? | |  | |  | |
| Does FVC meet ATS/ERS repeatability criteria? | |  | |  | |
| Has an appropriate technical comment and quality grade been added to the report? | |  | |  | |
| **Spirometry interpretation** | | **Candidate to complete**  **(indicate with** ✓/🗶) | | **Assessor’s comments**  Provide enough information to support the final assessment outcome | |
| The spirometric pattern identified from the final reported values is (indicate by ticking in the relevant box): | | | | | |
| Normal pattern | |  | |  | |
| Obstructive pattern | |  | |  | |
| Restrictive pattern (needs further investigation) | |  | |  | |
| Obstructive with reduced FVC pattern (needs further investigation) | |  | |  | |
| If the spirometry has an obstructive pattern, grade the degree of the obstruction based on current ATS/ERS interpretative criteria (indicate by ticking in the relevant box): | | | | | |
| Mild | |  | |  | |
| Moderate | |  | |  | |
| Moderately severe | |  | |  | |
| Severe | |  | |  | |
| Very severe | |  | |  | |
| Assessment of bronchodilator responsiveness test, based on current ATS/ERS guidelines (This section is only applicable to Candidates who have indicated that they perform pre- and post-bronchodilator assessments in their workplace): | | | | | |
| Non-significant bronchodilator response | |  | |  | |
| Significant bronchodilator responsiveness is demonstrated | |  | |  | |
| A significant improvement in FEV_1_ of_____mL and ____% | |  | |  | |
| A significant improvement in FVC of_____mL and ____% | |  | |  | |
| **Calibration/Calibration verification^#^** | | | | | |
| Has the calibration/calibration verification been performed and submitted for this test? | | |  | |  |
| Has a graph or tabulated data been submitted showing the calibration verification results for this test? | | |  | |  |
| Has evidence been provided for calibration verification at multiple flows? | | |  | |  |
| Has evidence of action been provided for out-of-range data? (if applicable) | | |  | |  |
| *# One calibration/calibration verification report can cover multiple client tests if performed on the same day or in the same testing session. Indicate this clearly on the calibration/calibration verification report submitted.* | | | | | |

**Portfolio Assessment Outcome:**

*Tick relevant assessment outcome*

| **Competency Achieved**  All competency elements completed |  |
| --- | --- |
| **Competency Not Achieved**  Re- assessment required |  |

| **Assessor Feedback:** |
| --- |
|  |
| **Recommendations for re-assessment** |
|  |

| **Name of Assessor: _____________________________** | **Date: _________** |
| --- | --- |
| **Signature: _____________________________________** |  |
| **Contact e-mail: _________­­­­­­___­­­­­_____________________________________** | |

**Appendix S10. Spirometry practical assessment**

The purpose of this assessment is to evaluate the Candidate’s ability to perform spirometry on a client and assess the result in accordance with current ATS/ERS standards. The Candidate will be observed performing a test on a client and assessed against the *Spirometry Practical Assessment Checklist.*  In addition, oral responses to a selection of *Spirometry Concept Questions* will be required to demonstrate that the Candidate understands important concepts underpinning the performance of quality spirometry.

Demonstration of the performance criteria listed in the checklist and answers to the concept questions will provide evidence of successful completion of the spirometry practical assessment. Detailed information is provided in S6.2.

- 1. **Spirometry practical assessment checklist**

| **Candidate name: _____________________________** | **Test date: _________** | | |
| --- | --- | --- | --- |
| **Assessor name: ______________________________** | **Test time: _________** | | |
| **Competency Elements and Performance Criteria** | **Achieved**  **Yes/**  **No** | **Assessor comments**  *Provide enough information to support the final assessment outcome* |  |
| **Pre-test Preparation** |  |  |  |
| Performs a calibration verification of the spirometer using a 3-L syringe |  |  |  |
| Correctly identifies the client (name, DOB and address) |  |  |  |
| Records correct demographic details (age, height, sex, ethnicity) |  |  |  |
| Identifies reason for test |  |  |  |
| Identifies contraindications |  |  |  |
| Identifies prior use of respiratory medication e.g. time inhalers were used, if applicable |  |  |  |
| Explains the test procedure clearly and comprehensively, including use of mouthpiece and nose peg |  |  |  |
| Applies infection control procedures |  |  |  |

| **Competency Elements and Performance Criteria** | | **Achieved**  **Yes/No** | | **Assessor’s comments**  *Provide enough information to support the final assessment outcome* |
| --- | --- | --- | --- | --- |
| **Test performance** | | **Pre** | **Post** |  |
| Big breath in to TLC | Encouraged inspiration to Total Lung Capacity (TLC) |  |  |  |
| Rapid Start | Without hesitation or back extrapolation |  |  |  |
| Free from interruption to flow | No cough, tongue occlusion or interruption to flow within the first second, or early termination |  |  |  |
| End of forced expiration criteria met | Volume plateau achieved and FET ≤ 15 seconds |  |  |  |
| Rapid IVC to TLC | Expiratory and Inspiratory volumes match (if performing flow-volume loop) |  |  |  |
| Gives suitable corrective instructions | Identifies when client is not meeting the test requirements and repeats instructions |  |  |  |
| **Assessment of results** | | **Pre** | **Post** |  |
| Appropriate number of technically acceptable trials performed | |  |  |  |
| Repeatability criteria were achieved for FEV_1_ | |  |  |  |
| Repeatability criteria were achieved for FVC | |  |  |  |
| Usability criteria were achieved for FEV_1_ and/or FVC | |  |  |  |
| **Bronchodilator responsiveness testing (if applicable)** | | **Achieved**  **Yes/No** | |  |
| Administration of bronchodilator to workplace protocols | |  | |  |
| Waits appropriate time before performing post-bronchodilator spirometry | |  | |  |
| **Final spirometry report** | |  | |  |
| Technical comments and grading of test quality | |  | |  |
| Relevant statement about prior medication use before test | |  | |  |
| Appropriate comment on bronchodilator dose and delivery method if performed | |  | |  |
| Report final data values appropriately | |  | |  |
| Identify spirometric pattern | |  | |  |
| Identify significant reversibility (percentage and volume change) (if applicable) | |  | |  |

| **Spirometry Concept Questions**  Please ask questions 1 and 2 and a further 5 of the following; if other questions are asked please write these into the empty rows below  *(Indicate the questions asked with a* ✓ *in the middle column)* | **Assessor's comments**  *Indicate clearly if a question is answered correctly/not answered correctly* |
| --- | --- |
| What does spirometry measure? |  |
| What problems with your spirometer could affect the measured results? |  |
| What is the impact of demographic data on the reference range? For example, selecting the wrong gender. |  |
| Can you list 2 contraindications to performing spirometry? |  |
| Outline infection control procedures (eg hand hygiene, handling of reusable and disposable medical devices, use of inline filters) |  |
| Are your results acceptable? What are the current ATS/ERS acceptability criteria for spirometry |  |
| Are your results repeatable? What are the current ATS/ERS repeatability criteria for spirometry |  |
| What is the impact of not reaching end of forced expiration test criteria on FEV_1_ and FVC? |  |
| If a client could not meet end of forced expiration criteria |  |
| If your results are not repeatable, what would you do? |  |
| You would usually do no more than how many blows? |  |
| How long should you wait before measuring post-bronchodilator spirometry? |  |
| What constitutes significant bronchodilator responsiveness |  |
| What units and conditions (ATPS vs BTPS) are used to report FEV_1_? |  |
| What is the required accuracy of a 3-litre calibration syringe? |  |
| Why do you calibrate a flow-based spirometer at different flows? |  |
| Do you perform biological control testing and, if so, briefly describe your protocol? |  |
| What are the criteria for selecting FEV_1_ and FVC for the final report? |  |
| What adverse events could occur during the test? |  |

**Practical Assessment Outcome:**

*Tick relevant assessment outcome*

| **Competency Achieved**  All competency elements completed, and questions answered correctly |  |
| --- | --- |
| **Competency Not Achieved**  Re - assessment required |  |

| **Assessor Feedback:** |
| --- |
|  |
| **Recommendations for re-assessment** |
|  |

| **Name of Assessor: _____________________________** | **Date: ________** |
| --- | --- |
| **Signature: ____________________________________** |  |
| **Contact e-mail: ________________________________________________** | |

**Appendix S11. References**

1. Graham BL, Steenbruggen I, Miller MR, Barjaktarevic IZ, Cooper BG, Hall GL, Hallstrand TS, Kaminsky DA, McCarthy K, McCormack MC, Oropez CE, Rosenfeld M, Stanojevic S, Swanney MP, Thompson BR. Standardization of Spirometry 2019 Update. An Official American Thoracic Society and European Respiratory Society Technical Statement. *Am J Respir and Crit Care Med* 2019; **200:** e70-e88.
2. Quanjer PH, Stanojevic S, Cole TJ, Baur X, Hall GL, Culver BH, Enright PL, Hankinson JL, Ip MS, Zheng J, Stocks J. Multi-ethnic reference values for spirometry for the 3-95-yr age range: the global lung function 2012 equations. Eur Respir J. 2012; **40**:1324-43.
3. Brazzale D, Graham H, Swanney MP. Reference values for spirometry and their use in test interpretation: A position Statement from the Australian and New Zealand Society of Science. Respirology. 2016; 2, 1201-1209.
4. Pellegrino R, Viego G, Brisasco V, Crapo RO, Burgos F, Casaburi R, Coates A, van der Grinten CPM, Gustafsson P, Hankinson J, Jensen R, Johnson DC, MacIntyre N, McKay R, Miller MR, Navajas D, Pedersen OF, Wanger J. Interpretative strategies for lung function tests. *Eur Respir J*. 2005; **26**: 948-968.
