# Supplementary material for: Assessing spirometry competence through certification in community‐based healthcare settings in Australia and New Zealand: A position paper of the Australian and New Zealand Society of Respiratory Science
Source: Respirology. 2020 Dec 14;26(2):147–52. doi: 10.1111/resp.13987 (PMC7898916; doi:10.1111/resp.13987)
Supplement: Supplementary file 2 — Visual Abstract Spirometry Certification: A pathway to improving practice in community‐based healthcare settings. [file RESP-26-147-s002.PDF]

ANZSRS
Australian and New Zealand Society of Respiratory Science Ltd
www.anzsrs.org.au

Apply for Community-based Spirometry Certification

Spirometry Certification:

A pathway to improving practice in community-based healthcare settings

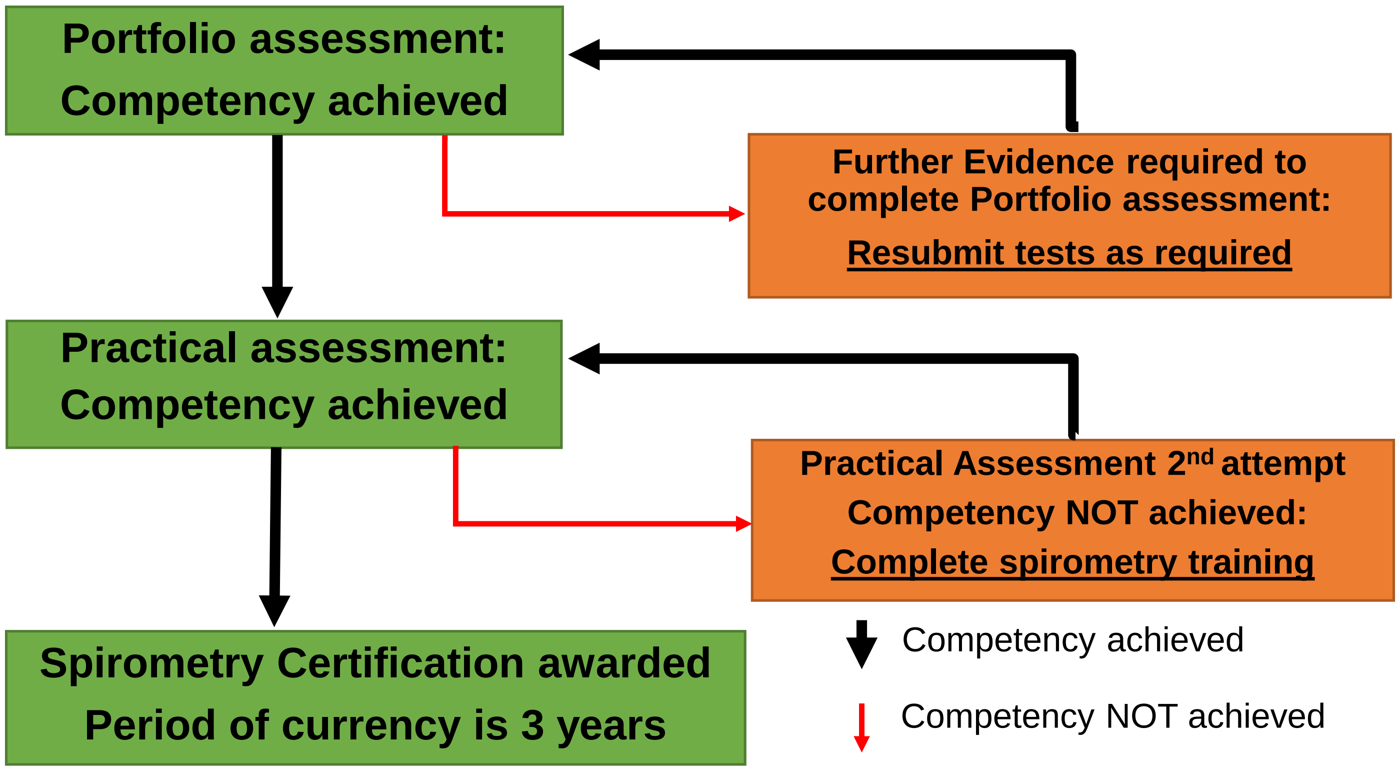

Respirology

Assessing spirometry competence through certification in community-based healthcare settings in Australia and New Zealand: A position paper of The Australian and New Zealand Society of Respiratory Science

Schneider et al., Respirology 2020 DOI: 10.1111/resp.13987
